# 

**Published:** 1841-11

**Authors:** 


### WESTERN JOURNAL

OF

### MEDICINE AND SURGERY.

### COLLABORATORS.

Edward H. Barton, M. D. *Professor of the Theory and Practice of Medicine, in the Medical College of Louisiana.*

William J. Barbee, M. D. *Cincinnati, Ohio.*

George W. Bayless, M. D. *Demonstrator of Anatomy in the Louisville Medical Institute.*

Solon Borland, M. D., *Memphis, Tennessee.*

A. H. Buchanan, M D. *Columbia, Tennessee.*

Charles Caldwell, M. D. *Professor of the Institutes of Medicine and Medical Jurisprudence in the Louisville Medical Institute.*

Samuel A. Cartwright, M. D. *Mississippi.*

A. Clapp, M. D. *New Albany, Indiana.*

Jedediah Cobb, M. D. *Professor of Anatomy in the Louisville Medical Insti*

Thomas W. Colescott, M. D. *Louisville, Ky.*

John Esten Cooke, M. D. *Professor of the Theory and Practice of Medicine in the Louisville Medical Institute.*

Samuel D. Gross, M. D. *Professor of Surgery in the Louisville Medical Institute.*

John Hardin, M. D. *Munfordsville, Ky.*

John P. Harrison, M. D. *late Professor of Materia Medica, in the Cincinnati College.*

John F. Henry, M. D. *Bloomington Illinois*

Samuel P. Hildreth, M. D. *Marietta, Ohio.*

Samuel Hogg, M. D. *Nashville, Tennessee.*

M. Z. Kreider, M. D., *Lancaster, Ohio.*

Moses L. Linton, M. D. *Springfield, Kentucky.*

Henry Miller, M. D. *Professor of Obstetrics and the Diseases of Women and Children in the Louisville Medical Institute.*

John W. Monette, M. D. *Washington, Mississsippi.*

Samuel B. Richardson, M. D., *Lecturer on Anatomy and Surgery, &c, Louisville, Ky.*

John L. Riddell, M. D. *Professor of Chemistry in the Medical College of Louisiana.*

Landon C. Rives, M. D. *late Professor of Obstetrics in the Medical Department of the Cincinnati College.*

Charles W. Short, M. D. *Professor of Materia Medica in the Louisville Medical Institute.*

G. Troost, M. D. *Professor of Chemistry and Mineralogy in the University of Nashville.*

Amasa L. Trowbridge, M. D. *Professor of Surgery, Willoughby University, Ohio.*

John A. Warder, M. D. *Cincinnati, Ohio.*

William Wood, M. D. *Cincinnati, Ohio*

### UNIVERSITY OF PENNSYLVANIA.

### MEDICAL DEPARTMENT.

### LOUISVILLE MEDICAL INSTITUTE.

The WESTERN JOURNAL OF MEDICINE and SURGERY is published monthly by the undersigned, at the corner of Main and Fifth streets, Louisville, at $5 per annum, payable in advance. Each number contains from 80 to 84 pages making two volumes in the year of about 500 pages.

Contributions to its pages by the Physicians of the Valley of the Mississippi, addressed to the Editors, are respectfully solicited.

Letters on business, to be addressed, postage paid, to the publishers.

☞Postmasters, by the regulations of the Postoffice Department, will frank letters containing subscription money, and all remittances so franked are at the risk of the publishers.

*July* 25, 1841. PRENTICE & WEISSINGER.

